# Is there a bidirectional relationship between allergic rhinitis and irritable bowel syndrome? A meta-analysis

**DOI:** 10.7189/jogh.15.04155

**Published:** 2025-06-13

**Authors:** Yifen Huang, Lun Cai, Jie Liu, RongRong Yang, Liping Wei, Xiongbin Gui, Huazheng Luo

**Affiliations:** 1 Department of Internal Medicine, The First Afﬁliated Hospital of Guangxi University of Chinese Medicine, Guangxi University of Chinese Medicine, Nanning, China; 2 Department of Rehabilitation, The First Afﬁliated Hospital of Guangxi University of Chinese Medicine, Guangxi University of Chinese Medicine, Nanning, China; 3 Department of Otorhinolaryngology, The First Afﬁliated Hospital of Guangxi University of Chinese Medicine, Guangxi University of Chinese Medicine, Nanning, China

## Abstract

**Background:**

Some studies suggest a link between allergic rhinitis (AR) and irritable bowel syndrome (IBS), but evidence is insufficient. This meta-analysis aimed to explore the relationship between AR and IBS.

**Methods:**

We searched the relevant literature in six electronic databases. We included a total of nine articles, seven of which took AR as the research object, two of which took IBS as the research object. We performed a meta-analysis using random effects and estimated the resultant odds ratio (OR).

**Results:**

A total of 10 627 patients with AR were included in seven studies, including 956 patients diagnosed with AR in the IBS population and 9671 patients diagnosed with AR in the non-IBS population. By heterogeneity test, *X*^2^ = 10.12, F-statistic (F) = 6, *P* = 0.12, *I^2^* = 41%, OR = 2.88, and Z-score (Z) = 21.97 (*P* < 0.00001), the results were statistically significant. Patients with AR have an increased risk of developing IBS compared to patients without AR. A total of 1099 patients with IBS were included in two studies, including 384 patients with IBS in AR patients and 715 patients with IBS in the healthy population. After the heterogeneity test, *X*^2^ = 0.11, F = 1, *P* = 0.74, *I^2^* = 0%, OR = 2.15, and Z = 11.81 (*P* < 0.00001), the results were statistically significant. Patients with IBS have an increased risk of developing AR compared to patients without IBS.

**Conclusions:**

The bidirectional association between AR and IBS provides a basis for exploring potential new mechanisms between the two.

**Registration:**

No. INPLASY202440057.

Allergic rhinitis (AR), also known as hay fever, is a type I hypersensitivity disease [1], which affects more than 400 million people worldwide [2]. The incidence is increasing year by year [3,4], and 34.5% of patients are still uncontrolled after treatment [5]. Similarly to AR, the global prevalence rate of irritable bowel syndrome (IBS) is as high as 15% [6], Both of these diseases seriously affect people's quality of life and increase the medical burden [7,8].

There is a potential relationship between AR and IBS in immune imbalance [9,10], intestinal flora disorder and mucosal barrier destruction [11,12]. In AR, a T helper 2 (Th2) dominant immune response is characterised by excessive Th2 cell activation and impaired Treg (T regulatory cells) function [13], Similarly, IBS patients exhibit elevated Th2 cytokine levels and suppressed Treg function in the gut [14]. Additionally, AR model mice have reduced intestinal flora diversity, with decreased levels of *Bifidobacterium* and *Lactobacillus*, which correlates negatively with serum Immunoglobulin E (IgE) levels [15]. In contrast, IBS patients experience gut flora imbalance that activates the immune response via the Toll-like receptor 4 pathway [16]. Regarding mucosal barriers, AR patients show increased nasal mucosal permeability, related to decreased expression of intestinal barrier proteins such as zonula occludens-1 [17]; the colonic mucosa of IBS model mice exhibits an epithelial barrier defect, which is associated with reduced zonula occludens-1 protein expression [18].These shared characteristics suggest an interactive mechanism between AR and IBS. supporting the hypothesis that they may act as risk factors for each other.

To test this hypothesis, we conducted two meta-analyses of different research objects to verify the correlation between AR and IBS. One was to collect the prevalence of AR in patients with IBS and non-IBS population for meta-analysis; the other was to analyse the prevalence of IBS in patients with AR and healthy individuals for mate analysis. The association between AR and IBS was verified from two analyses. Through these two analyses, we expect to be able to more clearly reveal the association between AR and IBS and provide a scientific basis for future research and clinical practice.

## METHODS

This meta-analysis adhered to PRISMA standards [19]. INPLASY has registered the meta-analysis protocol (Registration No. INPLASY202440057).

### Sources of literature

Search Chinese and English databases such as China National Knowledge Infrastructure, China Science and Technology Journal Database, the Chinese Biomedical Literature Service System, PubMed, PubMed Central, and Web of Science by computer. The words ‘Allergy,’ ‘Seasonal Allergic Rhinitis,’ ‘Pollen Allergy,’ ‘Hay Fever,’ ‘Gastrointestinal diseases,’ ‘Irritable Bowel Syndromes,’ ‘Irritable Colon,’ ‘Mucous Colitides,’ and ‘Pollinosis’ were used as Chinese and English search words. Combined English search format: ((Allergy or Seasonal Allergic Rhinitis or Pollen Allergy or Hay Fever or Pollinosis)) AND (Gastrointestinal diseases or Irritable Bowel Syndromes or Irritable Colon or Mucous Colitis). Full Chinese search words and format are provided in the **Online Supplementary Document** (Table S1 in the **Online Supplementary Document**). The retrieval period is from the self-built database of each database to from January 2000 to February 2024.

### Inclusion criteria

Studies were eligible for inclusion if they fulfilled the following conditions. First, the study was designed as a case-control or cohort study with a clear year of initiation or publication. Second, the study aimed to investigate the correlation between AR and IBS. Third, participants were diagnosed with AR if they met at least once of the following criteria: persistent clinical symptoms (*e.g*. nasal congestion, sneezing) occurring on ≥2 days per week for a duration of ≥4 weeks; positive results in allergen testing (skin prick test or serum IgE); consistency with the diagnostic criteria of the Allergic Rhinitis and its Impact on Asthma (ARIA) guidelines (2008 or 2016 edition); records of AR diagnosed at least once in the past five years were retrieved from national databases. Concurrent >2 diagnoses of AR documented in the previous 10 years. Fourth, the subjects were clinically diagnosed with AR attacks. The experimental group consisted of patients with IBS and the control group consisted of patients without IBS. The diagnosis of AR met the criteria; Fifth, participants were diagnosed with IBS met at least one of the following Rome criteria: Rome IV criteria: disease duration ≥6 months, with abdominal pain occurring at least one day per week over the past three months, and at least two of the following: Abdominal pain is associated with defecation. Abdominal pain episodes are accompanied by changes in bowel frequency. Abdominal pain episodes are accompanied by changes in stool characteristics. Rome III criteria: Abdominal pain or discomfort occurring at least three days per month over the past three months, and meeting at least two of the following: Symptoms are relieved after defecation. Episodes are accompanied by changes in bowel frequency. Episodes are accompanied by changes in stool characteristics. Rome II criteria: Abdominal pain or discomfort occurring for at least 12 weeks (not necessarily consecutive) over the past 12 months, and meeting at least two of the following: Symptoms are relieved after defecation. Episodes are accompanied by changes in bowel frequency. Episodes are accompanied by changes in stool characteristics. Sixth, the subjects of the study were clinically diagnosed patients with IBS attacks. The experimental group consisted of patients with AR, and the control group consisted of patients without AR. The patient's diagnosis of IBS met the criteria; Finally, studies were limited to Chinese and English documents.

### Exclusion criteria

Studies were excluded under the following circumstances. First, the literature did not mention explicit diagnostic criteria. Second, the establishment of an experimental group and a control group in the study did not meet the standards. Third, literature with incomplete sample data or incomplete information was excluded. Fourth, studies that focused on rhinitis caused by non-allergens, such as vasomotor rhinitis or drug-induced rhinitis, were excluded. Fifth, summary review articles, conference lecture papers, and republished literature were excluded. Sixth, studies involving drug or animal experiments were excluded. Seventh, non-clinical studies, including case-control studies, cohort studies, and cross-sectional studies, were excluded. Finally, studies that did not provide quantitative data on patients with AR and IBS were excluded.

### Screening and analysis of literature and data extraction

Two researchers (Huang Y and Luo H)used the same retrieval strategy to search and screen the literature independently according to inclusion and exclusion criteria. They read the titles and abstracts for preliminary screening and then read the full text for secondary screening. All selected literature met the inclusion criteria. If there was a dispute, discussions or third-party assistance were used. The main data extraction contents included the author, year, region, type of study, study population, age, sex, exposure factors, the sample size of the experimental group and control group, the number of patients with and without AR, and the number of patients with IBS and without IBS.

### Quality evaluation

The types of research in the included literature are mainly case-control studies and cohort studies. Therefore, the Newcastle-Ottawa Scale (NOS) was used to assess the quality of the included literature, and the studies were scored based on three aspects: selection of cases, comparability between groups, and measurement of exposure factors. The lowest score was 0, the highest score was nine, and the high quality of the literature was considered to be ≥6. The literature was independently evaluated by two researchers and if there was a disagreement, it was resolved by discussion or other researchers.

### Statistical analysis

In this study, the statistical software Review Manager5.4 (Cochrane Collaboration, Oxford, UK) was used to calculate the heterogeneity test, OR value, and 95% confidence interval (CI). The χ^2^ test was employed to analyse heterogeneity between studies. When *P* ≤ 0.1 and *I*^2^≥50%, indicating substantial heterogeneity, a random-effects model was selected, consistent with widely accepted interpretations [20] and Cochrane Handbook [21] recommendations, which suggest that *I*^2^ values of 50–90% may require a conservative analytical approach. If there is no significant heterogeneity, a fixed-effects model is used. Sensitivity analysis and subgroup analysis were also employed to examine the steady combined results and investigate potential causes of heterogeneity when the heterogeneity of test results was high.

## RESULTS

### Search result

**Figure 1:**
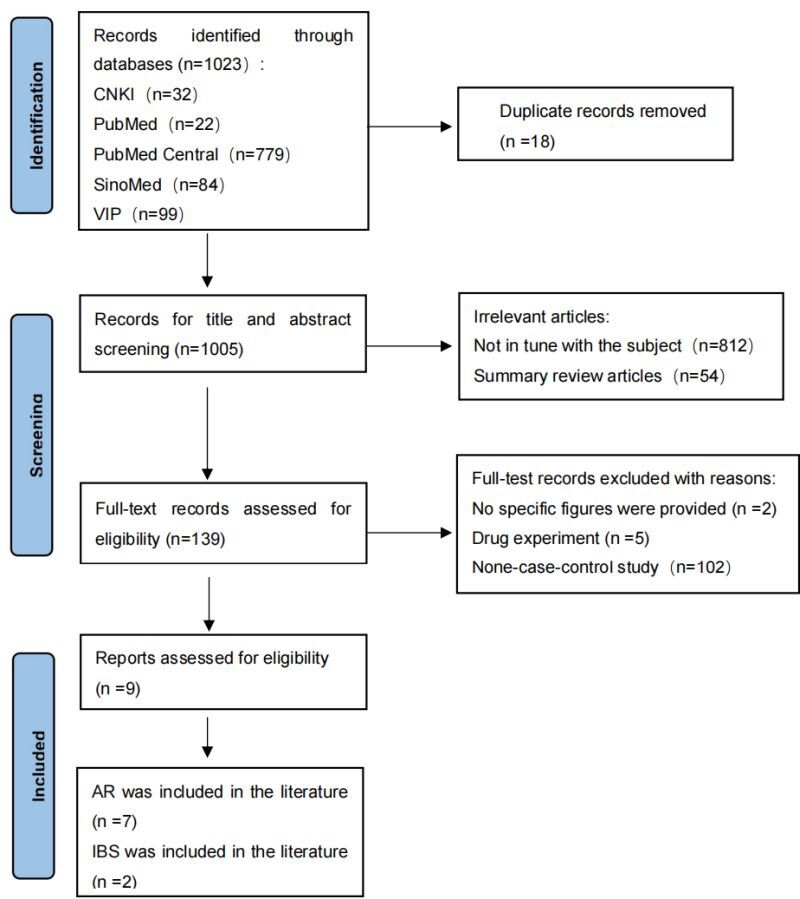
PRISMA flow diagram showing the selection of clinical studies included in the review.

This meta-analysis adhered to PRISMA standards [19]. International Platform of Registered Systematic Review and Meta-analysis Protocols (INPLASY) has registered the meta-analysis protocol (Registration No. INPLASY202440057). For the first time, 1023 articles were screened: 22 in Pubmed, 779 in Pubmed Central, seven in Web of Science, 32 in the China National Knowledge Infrastructure, 84 in the Wanfang Database and 99 in the Technology Journal Database. Endnote ×9 was used to delete 18 duplicates. After reviewing the titles and abstracts again, 139 articles were identified. These articles were carefully read, and upon detailed examination of the full text. According to the inclusion and exclusion criteria, five articles were excluded as experimental studies of drugs, two studies without specific data were excluded, 102 studies had no case-control study, 10 studies had no control group, and 11 studies were animal experiments, finally nine studies that met the criteria were included (**Figure 1**). Seven studies were included that took AR as research object [6,22–27] (**Table 1**), and two took IBS patients as research objects [28,29] (**Table 2**).

**Table 1:** The basic characteristics of AR as research objects were included

Author	Year	Region	Type of study	Total N of cases	Number of AR in IBS population (EG)	Number of AR in without IBS population (CG)	Age of (EG)	Age of (CG)	EG (male/female)	CG (male/female)	Diagnostic basis of IBS	Diagnostic basis of AR
M.P.Jones	2014	Europe	Cohort Study	1122	581	541	-	-	-	-	DDCR,RomeIII	DDCR
K.T.H.Siah	2020	Asia	Cross-Sectional Study	69	25	36	33.5	35.1	22/19	17/11	RomeIII,SPT (+)	Medical history, Physical examination, SPT (+)
S. Nybacka	2018	Europe	Cross-Sectional Study	77	66	11	31	27	59/164	18/29	RomeIII	Serum IgE levels, Specific allergen positive
C. Caffarelli	2007	Europe	Cross-Sectional Study	129	10	119	8.041	7.531	119/77	77/50	RomeIIstandard	Medical history, Physical examination, SPT (+)
M.C.Tobin	2008	America	Cohort Study	84	33	51	-	-	-	-	RomeII	Questionnaire survey
M. Kubo	2010	Asia	Case-Control Study	124	46	78	-	-	-	-	RomeIII	Questionnaire survey
G. Shalom	2017	Asia	Cross-Sectional Study	9030	195	8835	-	-	-	-	RomeIII	Clinical diagnosis

AR – Allergic rhinitis, CG – Control group, DDCR – Database diagnostic code retrieval, EG – Experimental group, IBS – Irritable bowel syndrome, SAR – Seasonal allergic rhinitis, SPT – Skin prick test, VMR – Vasomotor rhinitis

**Table 2:** The basic characteristics of IBS as research objects were included

Author	Year	Region	Type of study	Total N of cases	Number of IBS in AR population (EG)	Number of IBS in non-AR population (CG)		Age of EG	Age of CG	EG (male/female)	CG (male/female)		Diagnostic basis for IBS	Diagnostic basis of AR
S.W.Ho	2018	Asia	Cohort Study	1095	382	713	-	-		2998/4137		13528/14301	DDCR, >2 diagnoses of IBS in 10 y	DDCR, ≥2 diagnoses of AR over 10 y, and AR duration ≥180 d	
Z.Y.Fang	2018	Asia	Case-Control Study	4	2	2	-	-		-		-	RomeIV	ARIA(2008)	

AR – Allergic rhinitis, ARIA (2008) – Allergic Rhinitis and its Impact on Asthma in 2008, CG – Control group, DDCR – Database diagnostic code retrieval, EG – Experimental group, IBS – Irritable bowel syndrome, SPT – Skin prick test

### Results of the bias risk assessment included in the study

Most of the studies are case-control studies, so the NOS scale was used to evaluate the risk of bias independently by two researchers (**Table 3**).

**Table 3:** Results of the bias risk assessment included in the study

Author	Study population selection				Comparability	Result			Score
	**Representativeness of the exposed group**	**Method of selection of nonexposed group**	**Methods for determining exposure factors**	**Identify outcome indicators that should not be observed in the study**	**Whether the comparability of the exposed group and the exposed group is considered**	**Whether the research adequately evaluates the results**	**Results Whether follow-up was long enough after occurrence**	**Whether the exposed and non-exposed groups were adequately followed**	
C. Caffarelli	1	1	1	1	2	1	0	0	7
G. Shalom	1	1	1	1	2	1	0	0	7
M.P. Jones	1	1	1	1	2	1	0	0	7
M. Kubo	0	1	1	1	2	1	0	0	6
M. C. Tobin	1	1	1	1	2	1	0	0	7
S. Nybacka	1	1	1	1	2	1	0	0	7
S.W. Ho	1	1	1	1	2	1	0	0	7
Z.Y. Fang	1	1	1	1	2	1	0	0	7
K. T. H. Siah	1	1	1	1	2	1	0	0	7

In the included studies, the highest score was seven, the lowest was six, and the average score was 6.78. All the studies included populations can truly represent the characteristics of the exposure group; the nonexposed group and the exposed group are selected from the same population; exposure factors were determined by fixed archival records (*e.g*. searches of database diagnostic code) or structured interviews (*e.g*. questionnaires based on diagnostic criteria). The outcome indicators that must be observed were excluded at the beginning of the studies. The most important confounders were controlled for intergroup comparability. In outcome measures, follow-up or duration of follow-up was not mentioned in all included literature, so ‘sufficient follow-up after outcome occurrence’ and ‘adequate follow-up between exposed and nonexposed groups’ were subtracted from the assessment of bias risk. Since the self-report questionnaire was used as a basis for the diagnosis of AR, the representativeness of the two studies was poor [6,25].

### Results of the meta-analysis

**Figure 2:**
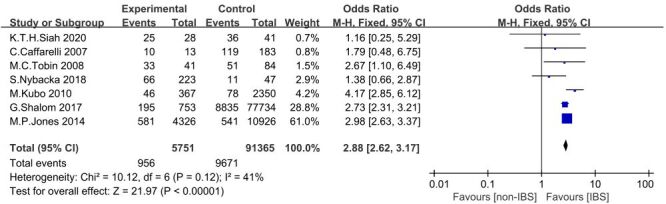
Forest plot showing the odds ratio with 95% confidence interval for the number of IBD with AR in the group and non-IBS with AR in nine studies. AR – allergic rhinitis, IBD – inflammatory bowel syndrome.

After the heterogeneity test, seven studies that took IBS as research object were included. A total of 10 627 AR patients were included, including 956 patients with AR in IBS patients and 9671 patients with AR in the non-IBS population. The results showed that *X*^2^ = 10.12, F = 6, *P* = 0.12, *I^2^* = 41%, OR = 2.88, and Z = 21.97 (*P* < 0.00001). The result was statistically significant. It appears that the proportion of AR is higher in patients with IBS than in non-IBS patients, patients with IBS appear to have an increased risk of developing AR (**Figure 2**).

**Figure 3:**
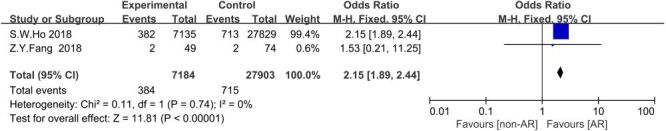
Forest plot showing the odds ratio with 95% confidence interval for the number of AR with IBS in the group and non-AR with IBS in nine studies. AR – allergic rhinitis, IBD – inflammatory bowel syndrome.

After the heterogeneity test, two studies were included that took AR as research object. There were 1099 IBS patients, including 384 IBS patients in the AR population and 715 IBS patients in the healthy population. The results showed that *X*^2^ = 0.11, F = 1, *P* = 0.74, *I^2^* = 0%, OR = 2.15, and Z = 11.81 (*P* < 0.00001), the results were statistically significant. It seems that the proportion of IBS is higher in patients with AR than in patients without AR, patients with AR appear to have an increased risk of developing IBS (**Figure 3**).

In order to explore the sources of heterogeneity among the included studies and the influence of different subgroup characteristics on the risk association of AR in IBS patients, subgroup analysis was performed according to the different Rome diagnostic criteria (Figure S1 in the **Online Supplementary Document**). Comparisons between the Rome II and Rome III subgroups revealed the following: Chi2 = 0.29, df = 1, *P* = 0.59, *I^2^* = 0%. These results suggest that different Rome diagnostic criteria yield similar assessments of the risk of AR in IBS patients. However, the heterogeneity within the Rome III subgroup (*P* = 0.05, *I*^2^ = 58%) indicates that it may primarily stem from differences among studies within this subgroup. During sensitivity analysis, we observed a significant reduction in heterogeneity after excluding the study by Nybacka et al [24] (*I*^2^ decreased from 41 to 18%, and the *P*-value increased from 0.12 to 0.30). This may be the excluded study included a variety of atopic diseases (*e.g*. eczema, asthma), which likely weakened the association strength between AR and IBS and caused its results to deviate from those of other studies.

In addition, subgroup analysis of different diagnostic criteria for AR was also performed (Figure S2 in the **Online Supplementary Document**). The comparison between the subgroup based on clinical symptoms and allergen detection and the subgroup based on database diagnostic code showed the following: Chi2 = 0.79, df = 1, *P* = 0.37, and *I^2^* = 0%. These results suggest that different AR diagnostic criteria provide similar assessment of AR risk in IBS patients. However, the heterogeneity within subgroups according to clinical symptoms and allergen detection (*P* = 0.08, *I*^2^ = 48%) suggests that it may be due primarily to differences between studies within that subgroup. During the sensitivity analysis, we observed a significant reduction in heterogeneity after excluding the study by M.Kubo et al. [25] (*I*^2^ decreased from 41% to 22%, *P*-value increased from 0.08 to 0.27). This may be attributed to the fact that the excluded studies Kubo study focused on Japanese adults, while other literatures were mostly based on European and American populations. The genetic background, dietary habits and environmental exposure of Asian population may lead to the differences in the association between IBS subtypes and allergy. However, some included studies lacked information on age and gender, which limited further analysis of potential confounding factors.

## DISCUSSION

### Description of main research results

This meta-analysis demonstrates a close relationship between AR and IBS. The proportion of AR among IBS patients is higher than among non-IBS patients, suggesting an increased risk of AR in those with IBS; conversely, the proportion of IBS among AR patients is higher than among non-AR patients, indicating an increased risk of IBS in those with AR. These results are consistent with previous studies. One study found that the incidence of AR patients had a 40% increased risk of IBS compared with non-AR patients [30], and children with gastroenteritis in early life, such as IBS, have a 1.49-fold higher risk of developing AR later in life than children without early gastroenteritis [31]. This evidence supports a bidirectional relationship between the two conditions. Furthermore, there are numerous potential mechanisms associated with this comorbidity.

Patients with AR have a higher risk of IBS than those without AR. It may be related to the change of intestinal microbial level and the increase of mast cell activity and number. Studies have shown that AR patients exhibit significantly reduced gut microbial diversity [32,33], characterised by decreased abundance of butyrate-producing bacteria (*e.g. Clostridiales*) and elevated *Bacteroidetes* levels [34]. These microbial shifts may impair intestinal barrier integrity and promote systemic Th2 inflammation, potentially amplifying mast cell-mediated allergic responses. Jenny Magnusson et al. found that mast cells and eosinophils were increased in the duodenal tissue of patients with seasonal AR during the birch pollen season, and mast cell increase may promote visceral hypersensitivity, disrupt the function of the epithelial barrier, and interact with the gut microbiota. Results in the occurrence of IBS [11,35]. Another study also showed that increased mast-cell activity, release of soluble mediators (*e.g*. trypsin), and improved paracellular permeability of the colon in patients with previous allergic diseases can lead to more severe symptoms in patients with IBS [36], which are alleviated by immune agents [37]. Kim et al. found that probiotics isolated from human faeces (*e.g. Lactobacillus* (a sub bacterial taxon belonging to Bacilli) could restore Th2/ Treg cells imbalance by inhibiting the differentiation of Th cells into Th2 cells and inducing differentiation into Treg cells) and modulation of the gut microbiota (inhibiting the subordinate bacterial taxon composition of Actinobacteria) to alleviate the symptoms of AR [38].

### Differences from previous studies and implications

Previously, we investigated the risk of AR in patients with inflammatory bowel disease (IBD) [39]. Both studies examined the association between diseases of the gastrointestinal system and AR in a meta-analysis of data collected from databases, and both IBD and IBS may share symptoms, such as abdominal pain and diarrhoea [40]. In fact, this meta-analysis is different from our previous study. First, in this meta-analysis, we found that IBS patients have an increased risk of developing AR and that AR may also be an early warning factor for IBS. In contrast, in the previous meta-analysis, we only validated the higher risk of AR in IBD patients. Second, IBS and IBD are different diseases, and IBD is a chronic intermittent disorder characterised by intestinal inflammation, including Crohn's disease and ulcerative colitis. A condition known as IBS is characterised by gastrointestinal symptoms such as abdominal pain, constipation, and diarrhoea. However, the pathogenesis of IBS is still unclear, and there are no etiologic anatomical or biochemical abnormalities that can be used to diagnose IBS [41]. Both have a strict diagnostic basis, although IBS-like symptoms occur in 35% of patients during remission of IBD and are confirmed by additional laboratory tests and imaging studies [40].

In terms of gut microbiota, Casen [42] found that the dominant bacteria in IBD cohorts were Proteobacteria (*Escherichia-Shigella*) and *Faecalibacterium prausnitzii* (*F. prausnitzii*), Bacteroides and *Prevotella*. *Firmicutes* (Bacilli), *Escherichia-Shigella*, Actinobacteria and *Ruminococcus gnavus* were the dominant bacteria causing dysbiosis in the IBS cohort. There were differences in the dominant flora between the two cohorts.

Among them, *F. prausnitzii* is the dominant flora in IBD cohort (the abundance of flora is decreased relative to healthy people), and butyrate produced by *F. prausnitzii* maintains T helper 17 (Th17)/Treg balance by inhibiting histone deacetylase 1 [43]. It was found [44] that intragastric administration of *F. prausnitzii* and *Bacteroides faecis* (one of the bacteria in Bacteroides) in mice with inflammatory colitis could regulate the balance of Th17 and Treg-related cytokines (such as Interleukin-10 (IL-10) and Interleukin-17A (IL-17A)) in plasma. Increasing the ratio of Th17/Treg can alleviate the clinical symptoms and histological damage of inflammatory colitis model mice. There is evidence of functional antagonism between Th17 and Treg cells, where Treg cells play an important role in maintaining immune homeostasis [45] and Th17 cells are involved in the initiation of autoimmune and inflammatory diseases [46]. Immune tolerance and stability within the body are collaboratively regulated by Treg and Th17 cells, and these cells play a significant role in the pathogenesis and progression of AR [47]. Studies have found that the Th17/Treg ratio in peripheral blood of AR patients is lower than that of healthy controls, and the serum concentrations of IL-17, IL-10 cytokines and IgE are significantly higher than those of healthy controls, and IL-17 is positively correlated with total IgE level [48]. In conclusion, we dare to speculate that IBD causes the imbalance of intestinal flora, which breaks the balance of Th17/Treg and related inflammatory cytokines, leading to the imbalance of immune homeostasis and the possibility of inducing AR.

*Ruminococcus gnavus* was similarly found to be enriched in the gut of IBS patients in another study, with a positive correlation with IBS-related viral operational taxonomic units [49]. Studies have shown that *Ruminococcus gnavus* can activate intestinal Th2 immune response, promote the release of IL-4, IL-5, IL-13 and other cytokines, and drive mast cell activation and eosinophil recruitment, which is directly related to nasal inflammation of AR [50]. Elevated 5-Hydroxytryptamine (5-HT) levels in both AR and IBS patients may serve as a shared mediator. Lixiang Zhai [51] colonised *Ruminococcus gnavus* alone in sterile decimal and found that *Ruminococcus gnavus* could produce phenethylamine and tryptamine by decomposing phenylalanine and tryptophan in the diet. These substances directly stimulate the biosynthesis of 5-HT in the intestine, thereby increasing intestinal motility, leading to the occurrence or exacerbation of IBS symptoms. Moreover, in the cohort of IBS patients, faecal phenylethylamine and tryptamine were positively correlated with serum 5-HT and IBS symptoms. Derived from tryptophan, 5-HT is an important neurotransmitter that plays a crucial role not only in regulating intestinal motility and hypersensitivity functions but also in modulating immune responses [52], and hypersensitivity functions [53]. Studies have found that 5-HT in the serum of AR patients is higher than that of healthy controls [54],while 5-HT can induce IL-6 and IL-21 production by dendritic cells, promote the transformation of Treg to Th17 cells [55],destroy the balance of Treg and Th17, and lead to AR. Inhibition of 5-hydroxytryptamine 1B/1D receptor (5-HT1B1D) receptor activation [56] can reduce the levels of IL-4, serum IgE, and inducible nitric oxide synthase protein expression in AR model mice, thereby alleviating allergic reactions in AR mice.

*Escherichia-Shigella* was one of the top five dysbiosis flora in IBS and IBD, and the abundance of bacteria was increased compared with that of healthy people, indicating the overlap of intestinal flora in IBS and IBD. Studies have shown that excessive proliferation of *Escherichia-Shigella* can activate intestinal lamina propria immune cells and promote their release of proinflammatory cytokines IL-6 and Tumor Necrosis Factor-alpha (TNF-α), which can damage the intestinal mucosal barrier and further worsen IBS [57,58]. In addition, the relative abundance of *Escherichia-Shigella* in the faecal samples of AR model mice was higher than that of healthy control group. The imbalance of intestinal flora in AR model mice induced by vancomycin may lead to the increase of *Escherichia-Shigella*. It limits the production of Short-chain fatty acids (SCFA), aggravates the destruction of intestinal mucosal barrier, and aggravates AR [59]. In addition, SCFA plays an important role in intestinal physiological function [60], and SCFA depletion inhibits 5-HT synthesis by enterochromaffin cells, leading to abnormal regulation of intestinal function by the central nervous system, manifested as IBS-associated abdominal pain and altered bowel habits [61,62].

In terms of genetic inheritance, a recent meta-analysis of genome-wide association studies identified 163 IBD susceptibility loci. Many of these genes, such as Nucleotide-binding oligomerisation domain containing 2 (NOD2), Immune-related GTPase family M (IRGM), Autophagy-related 16-like 1 (ATG16L1), and Interleukin-23 receptor (IL23R), are involved in host-microbiota interactions [63]. The first gene identified to be associated with susceptibility to Crohn's disease was NOD2[64]. It leads to transcription of proinflammatory cytokine and chemokine genes, impairs intestinal immune defence and mucosal barrier establishment [65]and patients carrying heterozygous NOD2/ Caspase Recruitment Domain-containing Protein 15 (CARD15) mutations have a two-to 4-fold increased risk of Crohn's Disease (CD) compared with the general population [66]. Studies have shown that astragalus polysaccharides may improve rhinitis symptoms and reduce cytopathological changes in AR model rats by inhibiting NOD2-mediated Nuclear Factor kappa-light-chain-enhancer of activated B cells (NF-κB) activation [67]. In conclusion, NOD2 gene mutations are associated with the pathogenesis of IBD and AR.

A meta-analysis of genome-wide association studies of IBS identified 10 IBS risk loci. Combined with the Polygenic Risk Score (PRS)-based Phenome-Wide Association Study (PheWAS) approach, potential pleiotropic associations were found between IBS and 9 traits, including Proline-Rich Coiled-Coil 2A (PRRC2A), Constitutive Photomorphogenesis 1 (COP1), Cell Adhesion Molecule 2 (CADM2), Low-Density Lipoprotein Receptor-Related Protein 1B (LRP1B), Sodium-Dependent Glucose Transporter 1 (SUGT1), Mediator Complex Subunit 12-Like (MED12L), Purinergic Receptor P2Y14 (P2RY14), Plant Homeodomain Finger 2 (PHF2), and Shisa Family Member 6 (SHISA6). No genes have been implicated in the pathogenesis of AR, but CADM2 and PHF2 have been implicated in anxiety disorders [68], depression, [69] and autism [70]. Mood and anxiety disorders are associated with an increased risk of AR. Patients with IBS have been found to have more severe symptoms of depression and anxiety than those with IBD [71], and AR is associated with a significantly increased risk of depression, anxiety, stress, and suicide attempts [72], which can act as a stressor and further lead to worsening IBS symptoms [73].

### Study heterogeneity

#### Differences in diagnostic criteria for IBS

Different Rome criteria (II/III/IV) and AR diagnostic criteria were used in the included studies, and their requirements for duration and frequency of symptoms were significantly different. Although subgroup analysis showed that different Rome criteria (II/III/IV) and AR diagnostic criteria had no significant effect on the risk assessment of the association between AR and IBS, the differences in the definition of symptom severity and duration by different Rome criteria may lead to the heterogeneity of the study population. A study showed that 15% of 542 patients with IBS who met Roman III criteria did not meet Roman IV. The severity of IBS is more severe in patients who meet Roman III and IV criteria [74]. The inconsistent application of the Rome diagnostic criteria may affect the accuracy of the number of patients with IBS, which may lead to differences between the probability of AR in IBS patients and the actual probability.

#### Differences in AR diagnostic methods

In the diagnosis criteria of AR, some studies used the ARIA guideline criteria [29] or specific positive allergens [24], while others used medical history combined with a skin prick test [22,75] or a self-reported questionnaire [6,25]. These different diagnostic criteria may also affect the outcome of the study. In addition, the methods of allergen detection in the included studies were different, and patients with non-IgE-mediated localised AR may have been missed.

#### Diversity of study designs

The included studies included cohort studies, cross-sectional studies and case-control studies, and different study designs may introduce bias. Cross-sectional studies are susceptible to recall bias, while cohort studies can better verify the temporal relationship. design differences may weaken the strength of causal inferences.

### Advantages of this study

A meta-analysis of the relationship between AR and IBS was conducted for the first time. In addition to collecting the literature with AR as the research object, the literature with IBS as the research object was also collected. From these two mate-analyses, it was concluded that the two diseases were warning factors for each other. The inclusion and exclusion criteria were strict and the quality assessment of the literature suggested that the quality of inclusion was relatively high, which further confirmed the reliability of the results.

### Study limitations and generalisability

Our study has several limitations that may influence the interpretation and applicability of the results. First, language restrictions were imposed due to the research team's proficiency and the predominant use of standardised diagnostic criteria in Chinese and English. This limitation may have resulted in the exclusion of high-quality studies published in other languages. Second, the included studies were primarily from Europe and Asia. Given the differences in genetic background, allergen distribution, and medical resources, the findings may not be generalisable to regions outside of Europe and Asia, potentially limiting the global applicability of our results. Finally, most of the included studies lacked detailed data on age, gender, and psychological comorbidities, which restricted our ability to conduct further analysis of potential confounding factors.

### Implications for clinical practice

This study indicated a close relationship between IBS and AR. They are mutual risk factors and have many common pathogeneses. When a patient presents a certain medical condition, we need to assess their risk of developing the other medical condition. When an effective treatment for a particular medical condition emerges, we can attempt to use it for another medical condition that shares a common pathogenesis.

### Implications for future research

First, we carry out relevant large sample studies to reduce sampling error. Standardising relevant studies and adopting unified diagnosis and evaluation criteria for diseases to improve the level of research design and make the resulting studies more credible. For example, exploring the correlation between different types of AR or specific allergens (such as pollen, dust, mites, *etc*.) and the incidence of IBS, or exploring studies on the correlation between different types of IBS (constipation or diarrhoea) and AR. Second, the relationship between AR and IBS can be further deepened by developing the corresponding drugs through the comorbidity mechanism of the two diseases and exploring the effect of drugs on the treatment of the two diseases. Third, both IBD and IBS can increase the risk of AR through distinct characteristics in intestinal flora, genetic profiles, immune responses, and the 5-HT energetic pathway.

## CONCLUSIONS

The results of this study suggest that IBS and AR may be risk factors for each other. It also suggests that allergic diseases are not only associated with gastrointestinal diseases, but also may be associated with immune-mediated diseases. This study provides a basis for exploring AR, IBS, and the existence of new mechanisms between them. In clinical work, this can guide physicians in evaluating the risk of AR in IBS or IBS in patients with AR.

## Additional material


Online Supplementary Document
