# Supplementary material for: Is there a bidirectional relationship between allergic rhinitis and irritable bowel syndrome? A meta-analysis
Source: J Glob Health. 2025 Jun 13;15:04155. doi: 10.7189/jogh.15.04155 (PMC12163863; doi:10.7189/jogh.15.04155)

Supplement to: Huang Y, Cai L, Liu J, Yang R, Wei L, Gui X, Luo H. Is there a bidirectional relationship between allergic rhinitis and irritable bowel syndrome? A meta-analysis. J Glob Health. 2025;15:04155.

Table S1 in the Online Supplementary Document. Chinese search words and format

Category	Terms
Allergy-related words	“过敏” “ 过敏性鼻炎” “变应性鼻炎” “常年性鼻炎” “变态反应性鼻炎” “常年变应性鼻炎” “常年性变应性鼻炎” “常年性过敏性鼻炎” “常年性变态反应性鼻炎”
IBS-related words	“胃肠道疾病” “肠易激综合症” “大肠激燥症” “肠道易激综合症”“肠道易激综合征” “肠易激综合征” “肠易激惹综合征” “肠应激综合征”
Chinese search format	（（（过敏）or（过敏性鼻炎）or（变应性鼻炎）or（常年性鼻炎）or（变态反应性鼻炎）or（常年变应性鼻炎）or（常年性变应性鼻炎）or（常年性过敏性鼻炎）or（常年性变态反应性鼻炎））and（（胃肠道疾病）or（肠易激综合症）or（大肠激燥症）or（肠道易激综合症）or（肠道易激综合征）or（肠易激综合征）or（肠易激惹综合征）or（肠应激综合征））

Figure S1. Forest plot showing subgroup Analysis by Rome Criteria (III vs IV) for the number of AR with IBS in the group and non-AR with IBS in nine studies. AR – allergic rhinitis, IBD – inflammatory bowel syndrome.

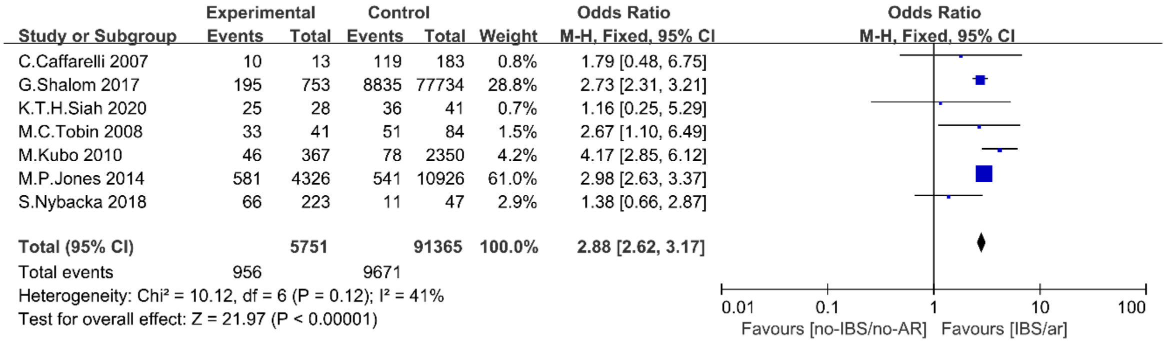

Figure S2. Forest plot showing subgroup Analysis by AR Criteria or the number of AR with IBS in the group and non-AR with IBS in nine studies. AR – allergic rhinitis, IBD – inflammatory bowel syndrome.

Study or Subgroup	Experimental Events	Experimental Total	Control Events	Control Total	Weight	Odds Ratio M-H, Fixed, 95% CI
**6.4.1 Symptoms and allergen testing**						
C.Caffarelli 2007	10	13	119	183	0.8%	1.79 [0.48, 6.75]
G.Shalom 2017	195	753	8835	77734	28.8%	2.73 [2.31, 3.21]
K.T.H.Siah 2020	25	28	36	41	0.7%	1.16 [0.25, 5.29]
M.C.Tobin 2008	33	41	51	84	1.5%	2.67 [1.10, 6.49]
M.Kubo 2010	46	367	78	2350	4.2%	4.17 [2.85, 6.12]
S.Nybacka 2018	66	223	11	47	2.9%	1.38 [0.66, 2.87]
**Subtotal (95% CI)**		**1425**		**80439**	**39.0%**	**2.73 [2.36, 3.16]**
Total events	375		9130			
Heterogeneity: Chi² = 9.69, df = 5 (P = 0.08); I² = 48%						
Test for overall effect: Z = 13.36 (P < 0.00001)						
**6.4.2 Database diagnostic codes**						
M.P.Jones 2014	581	4326	541	10926	61.0%	2.98 [2.63, 3.37]
**Subtotal (95% CI)**		**4326**		**10926**	**61.0%**	**2.98 [2.63, 3.37]**
Total events	581		541			
Heterogeneity: Not applicable						
Test for overall effect: Z = 17.40 (P < 0.00001)						
**Total (95% CI)**		**5751**		**91365**	**100.0%**	**2.88 [2.62, 3.17]**
Total events	956		9671			
Heterogeneity: Chi² = 10.12, df = 6 (P = 0.12); I² = 41%						
Test for overall effect: Z = 21.97 (P < 0.00001)						
Test for subgroup differences: Chi² = 0.79, df = 1 (P = 0.37), I² = 0%						

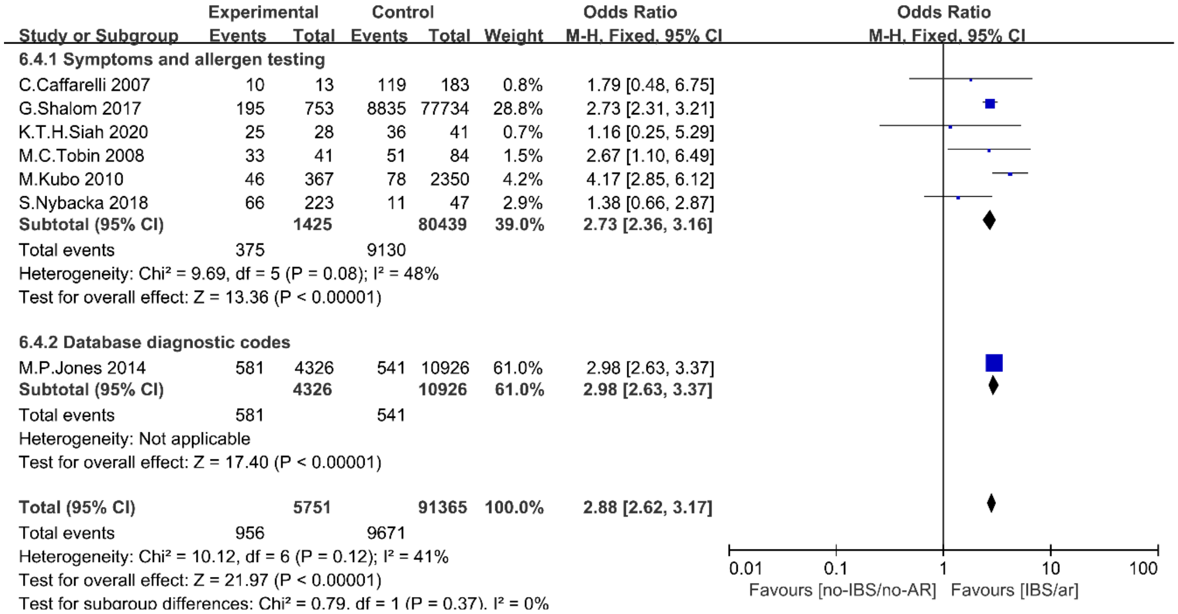

Supplement: Online Supplementary Document [file jogh-15-04155-s001.pdf]
